# Interactomics profiling of the negative regulatory function of carbon monoxide on RANKL-treated RAW 264.7 cells during osteoclastogenesis

**DOI:** 10.1186/1752-0509-8-57

**Published:** 2014-05-18

**Authors:** Feng-Jen Tseng, Wei-Tso Chia, Jia-Fwu Shyu, Guo-Hau Gou, Huey-Kang Sytwu, Ching-Wu Hsia, Min-Jen Tseng, Ru-Yu Pan

**Affiliations:** 1 Graduate Institute of Medical Science, National Defense Medical Center, Neihu 114, Taipei, Taiwan, Republic of China; 2 Department of Orthopedics, Hualien Armed Force Hospital, Hualien 971, Taiwan, Republic of China; 3 Department of Health, Hsin Chu General Hospital, Hsinchu 300, Taiwan, Republic of China; 4 Department of Biology and Anatomy, National Defense Medical Center, Neihu 114, Taipei, Taiwan, Republic of China; 5 Department of Orthopaedics, Tri-Service General Hospital, National Defense Medical Center, Neihu 114, Taipei, Taiwan, Republic of China; 6 Department of Life Science, National Chung Cheng University, 168 University Road, Minhsiung, Chiayi 621, Republic of China

**Keywords:** Carbon monoxide, Osteoclastogenesis, Interactome, RANKL, RAW 264.7

## Abstract

**Background:**

During osteoclastogenesis, the maturation of osteoclast (OC) progenitors is stimulated by the receptor activator of nuclear factor-κB ligand (RANKL). Excess OC production plays a critical role in the pathogenesis of inflammatory bone disorders. Conversely, the inhibition of abnormal OC proliferation reduces inflammation-induced bone loss. Low concentrations of carbon monoxide (CO) are known to decrease inflammation and OC-mediated bone erosion but the molecular mechanism is unknown.

**Results:**

To obtain insight into the biological function of CO, cultured RANKL-treated RAW 264.7 cells were used in an in vitro experimental model of osteoclastogenesis. The results showed that CO inhibited: 1) tartrate-resistant acid phosphatase (TRAP)-positive cell formation; 2) F-actin ring production; 3) c-fos pathway activation; 4) the expression of cathepsin K, TRAP, calcitonin receptor, and matrix metalloproteinase-9 mRNAs; 5) the expression of nuclear factor of activated T cells, cytoplasmic, calcineurin-dependent 1 in translation. Protein-protein interaction analysis predicted mitogen-activated protein kinase kinase kinase 4 as the controlling hub.

**Conclusions:**

Low-concentrations of CO (250 ppm) may inhibit osteoclastogenesis. Data from STRING- and IPA-based interactome analyses suggested that the expression of proteins with the functions of signal transduction, enzymes, and epigenetic regulation are significantly altered by CO during RANKL-induced osteoclastogenesis. Our study provides the first interactome analysis of osteoclastogenesis, the results of which supported the negative regulation of OC differentiation by CO.

## Background

Bone homeostasis is strictly regulated through a dynamic balance between osteoblastogenesis (bone formation) and osteoclastogenesis (bone resorption) [1-5]. In the former, osteoblasts (OBs) control bone formation through the synthesis of bone matrix proteins. In osteoclastogenesis, large multinucleated osteoclasts (OCs) remove the mineralized matrix of bone tissue, resulting in bone resorption. OCs are derived from hematopoietic precursors of the monocyte-macrophage lineage, and their differentiation is regulated by macrophage colony-stimulating factor (M-CSF) [6]. Mutations in the M-CSF gene induce defects in the formation of macrophages and OCs, which suggests that immune cells and bone cells are derived from the same progenitors [7].

The receptor activator of nuclear factor (NF)-κB ligand (RANKL), a member of the tumor necrosis factor (TNF) family, regulates OC maturation and differentiation [8,9]. Bone-forming OBs express RANKL as do activated T cells which indicates a role for the immune system in osteoclastic bone resorption [9,10]. In addition, many inflammatory cytokines are known to modulate RANKL expression, including TNF-α [9,11], and the RANKL dependence of several inflammatory bone diseases has been reported [10]. Bone dysfunction may also arise following the production of RANKL by activated T cells, by directly triggering osteoclastogenesis [12].

Osteoclasts express the receptor activator of NF-κB (RANK), a type I membrane protein. Thus, the RANKL/RANK signaling cascade regulates not only the maturation of OC progenitors, but also the activity of OCs in normal bone remodeling [13,14]. Given the critical functions of OCs, an understanding of the process that control their differentiation is required for the successful treatment of many bone pathologies.

Carbon monoxide (CO) is an invisible and odorless gas with a binding affinity for heme that is 240 times higher than that of oxygen. Concentrations of CO in the air exceeding 3% (30000 ppm) are usually lethal [15,16]. CO is endogenously produced by mammals, by the degradation of heme, albeit in very low concentrations [17,18]. Interestingly, however, in small amounts (250 ppm) CO exhibits anti-apoptosis, anti-proliferation, anti-inflammatory, and many other biological activities [19-22]. For example, CO-releasing molecule-2 dose-dependently inhibits RANKL-induced osteoclastogenesis [23].

Mouse leukemic monocyte macrophage RAW 264.7 cells are derived from Abelson-virus-treated mice ascites and recognized as pre-OCs [24]. Following their stimulation with RANKL, these cells produce OCs in a pathway involving mitogen-activated protein kinases (MAPKs), extracellular-regulated kinase (ERK), p38, jun N-terminal kinase (JNK), and c-fos [25-27]. In this study, we used RANKL-treated RAW264.7 cells as an vitro model to investigate the effect of CO on the signaling pathway of RANKL-induced osteoclastogenesis. In addition, in an approach using interactomics to obtain a protein-protein interaction (PPI) network, we derived a genome-scale PPI map to deduce the potential signaling pathways and member proteins involved in the differentiation and activation of OCs, and the effects of CO on these pathways [28].

## Results

### Inhibition of RANKL-induced osteoclastogenesis in RAW 264.7 cells by low-dose CO without preventing growth or inducing apoptosis

**Figure 1:**
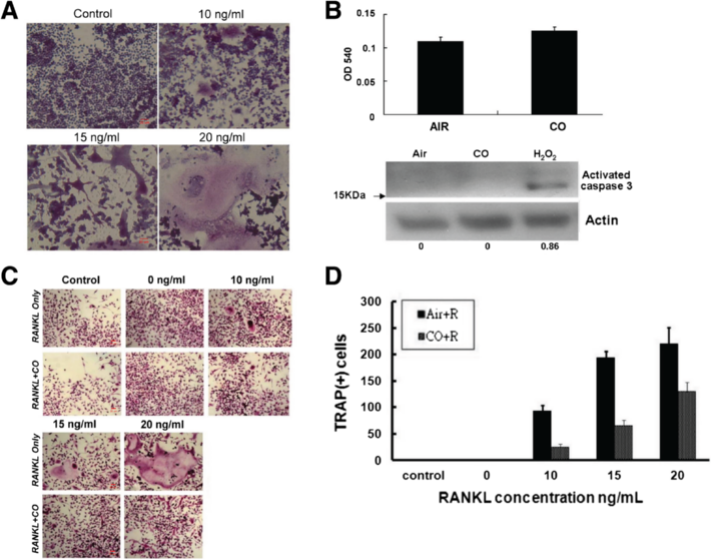
**CO reduces osteoclastogenesis by RANKL**-**treated RAW 264.7 cells in vitro. (A)** RAW 264.7 cells treated with 10, 15, and 20 ng RANKL/mL and examined by TRAP staining after 96 h. **(B)** Upper panel, RAW 264.7 cells exposed to air or 250 ppm CO for 96 h and then stained with 0.05% methylene blue, as described in Methods. The data are the mean from three experiments with triplicate samples; *bars*, +SD. Lower panel, Western blot analysis of activated caspase 3 expression by RAW 264.7 cells. **(C)** TRAP staining of RAW 264.7 cells treated with 20 ng RANKL/mL and then exposed to 250 ppm CO. **(D)** Bar chart showing the inhibitory effect of CO (250 ppm) on RAW 264.7 cells treated as in **(C)**.

RAW 264.7 cells incubated with 10, 15, and 20 ng RANKL/mL for 96 h gave rise to TRAP(+) multinucleated cells in a dose-dependent manner. When these RANKL-treated progenitor cells were exposed to 250 ppm CO, the formation of TRAP(+) multinucleated cells was inhibited by 73 + 5% (10 ng/mL), 70+ 5% (15 ng/mL), and 41+ 10% (20 ng/mL) (Figure 1C,D), We also check the effects of CO on osteoclastogenesis in bone marrow macrophages and the results are the same as RAW cells (Additional file 1). Moreover, after incubation with 250 ppm CO for 72 or 96 h, RANKL-treated RAW 264.7 cells retained their ability to proliferate and the expression of activated caspase-3, a marker of apoptosis, was not induced (Figure 1B).

### CO-induced inhibition of F-actin ring formation by osteoclasts

**Figure 2:**
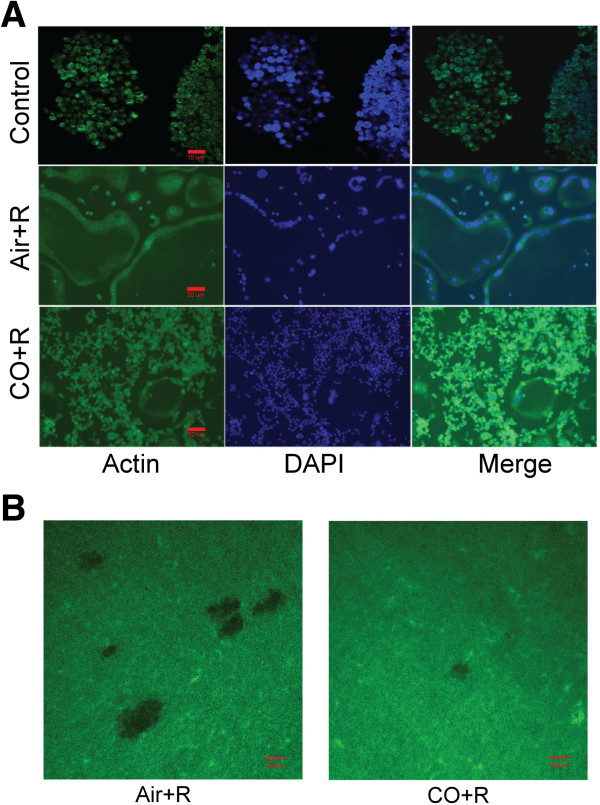
**CO inhibits F-actin ring formation by RANKL-treated RAW 264.7 cells. (A)** Effect of 250 ppm CO on the distribution of clustered F-actin filaments in RANKL-treated RAW 264.7 cells. Arrows show the position of the F-actin ring. **(B)** Pit formation assay of RANKL-treated RAW 264.7 cells cultured on dentin discs and incubated with air or CO.

Formation of the F-actin ring by OCs is a necessary step in bone resorption. As shown in Figure 2A and Figure 2B, in RAW 264.7 cells treated with 20 ng RANKL/mL and 250 ppm CO for 96 h, F-actin ring formation was reduced (Additional file 2). A similar inhibitory effect of osteoclast pit formation, an indicator of bone resorption, was observed on dentin discs in the CO but not the Air group.

### CO-induced suppression of RANKL-induced JNK and c-jun phosphorylation, and c-fos but not IκB-α expression

**Figure 3:**
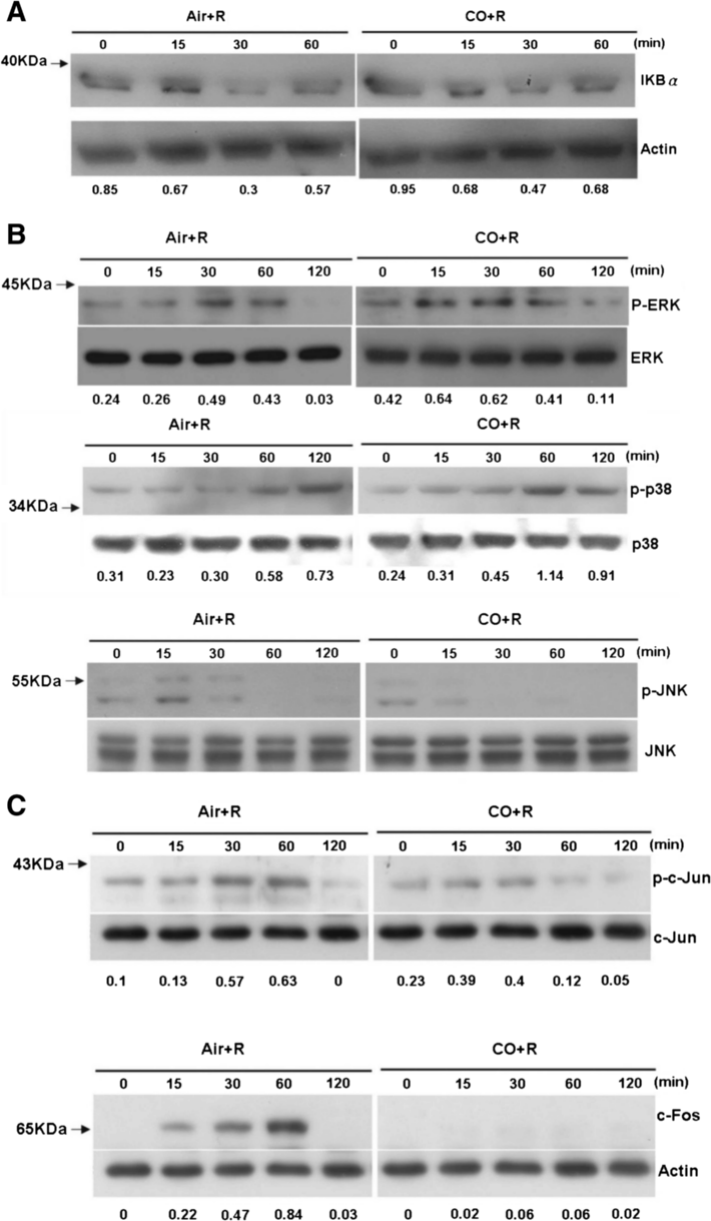
**CO suppresses JNK and c-jun phosphorylation and c-fos expression in RANKL-treated RAW 264.7 cells.** Western blots showing **(A)** the expression of IκB-α, as an indicator of NF-κB activity, **(B)** the phosphorylation of ERK, p38, and JNK, and **(C)** c-Jun and c-Fos expression.

The activation of NF-κB plays an important role in the differentiation of pre-OCs. As shown in Figure 3A, in RAW 264.7 cells exposed to 20 ng RANKL/mL and 250 ppm CO there was no change in the expression of IκB-α, which is normally induced by NF-κB and serves as its inhibitor. Furthermore, this same dose of CO inhibited the phosphorylation of RANKL-induced JNK and c-jun but not that of either ERK or mitogen-activated protein kinase p38 (p38) within 120 min (Figure 3B). The same concentration of CO down-regulated the expression of c-fos in similarly treated RAW 264.7 cells (Figure 3C).

### CO-induced reduction of *Acp5*, *Ctsk*, *Calcr*, *Mmp9*, and NFATc1 expression

**Figure 4:**
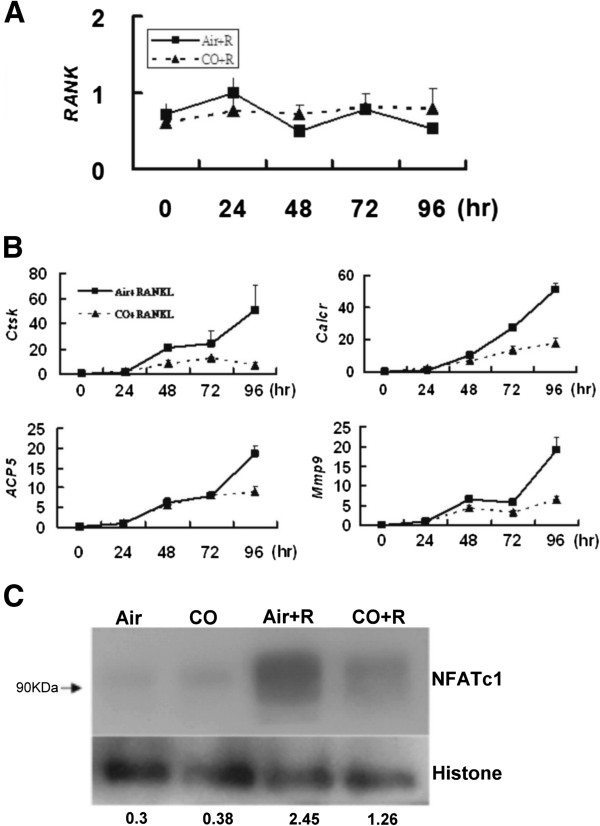
**CO reduces the expression levels of the genes *Acp5*, *Ctsk*, *Calcr*, *and Mmp9 *and of the protein NFATc1 but not that of RANK in RANKL-treated RAW 264.7 cells. (A)** Western blot analysis of RANK expression. **(B)** qRT-PCR analysis of *Acp5*, *Calcr*, *Cstk*, and *Mmp9* expression. **(C)** Western blot analysis of NFATc1 expression.

RANK is expressed on the cell surfaces of pre-OCs and OCs. As shown in Figure 4A, RANK mRNA expression in RAW 264.7 cells was not affected by 250 ppm CO within 96 h. Tartrate-resistant acid phosphatase (*Acp5*), the calcitonin receptor (*Calcr*), cathepsin K (*Cstk*), and matrix metalloproteinase-9 (*mmp9*) are unique markers of mature OCs. In RAW 264.7 cells treated with 20 ng RANKL/mL for 96 h, 250-ppm CO reduced the mRNA expression levels of all four genes (Figure 4B). Moreover, the expression of the protein nuclear factor of activated T cells, cytoplasmic, calcineurin-dependent 1 (NFATc1), which is the master regulator of osteoclastogenesis, was likewise significantly reduced (Figure 4C).

### Critical roles of MAP3K4 and the JNK signaling pathway in RANKL-induced osteoclastogenesis

**Figure 5:**
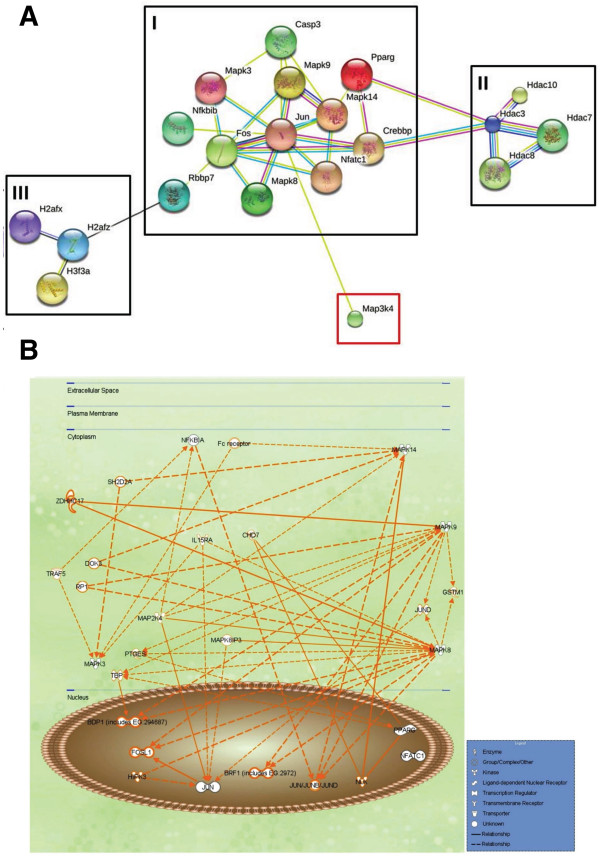
**The PPI network and signaling pathway analysis. (A)** The PPI network, as shown in the interaction view, generated by the STRING database. Black squares indicate the protein clusters, and the red square the controlling hub, MAP3K4. **(B)** The computed predicted signaling pathways that are activated in RANKL-treated RAW 264.7 cells.

The STRING database was used to construct a PPI map of RANKL-induced osteoclastogenesis. The map showed three protein clusters joined by mitogen-activated protein kinase kinase kinase 4 (MAP3K4) as a potential hub protein (Figure 5A). Using IPA software (see Methods), we identified the JNK protein family signaling pathway as a potential participant in the maturation of RAW 264.7 cells treated with 20 ng RANK/mL and exposed to 250 ppm CO (Figure 5B).

## Discussion

The potential benefits of CO have led to many studies of its possible therapeutic applications, which have been examined in different disease models. Sato et al. showed that CO prolonged the survival of mouse-to-rat cardiac grafts [29]. In mice with ischemic lung injury, the inhalation of CO was reported to prevent their death [30]. The protective effects of CO have also been demonstrated in animal models of autoimmune diseases [31-33].

Bone remodeling is a tightly regulated cycle involving the formation of bone by OBs and its resorption by OCs. The latter are monocyte-macrophage lineage-derived cells whose differentiation and maturation is regulated by OBs via RANKL. Excessive bone resorption, initiated by proinflammatory cytokines, also proceeds through RANKL and leads to abnormal bone destruction. Given the involvement of OCs in these processes, an understanding of their formation and function is crucial to prevent bone loss in inflammatory diseases such as arthritis.

In our study, small doses of exogenous CO not only reduced the degree of inflammation and the expression of RANKL. Consistent with these results, CO was shown to reduce joint destruction in a murine model of collagen-induced arthritis [34]. However, the effect of CO on RANKL-induced osteoclastogenesis has not been investigated. In this study, the differentiation of pre-OC murine macrophage-like RAW 264.7 cells into multinucleated OCs was stimulated using recombinant RANKL protein. This system was then used to examine the potential role of CO in osteoclastogenesis. In these experiments, the expression of several OC markers was examined, including the glycosylated monomeric metalloenzyme TRAP, which is expressed at high levels by. differentiated OCs. In the presence of 250 ppm CO, TRAP expression by RANKL-stimulated RAW 264.7 cells that had differentiated into TRAP (+) multinucleated OCs was blocked (Figure 1). Cytoskeletal reorganization in mature functioning OCs, including the formation of dot-like F-actin rings, was likewise inhibited in RANKL-treated RAW 264.7 cells, whereas their growth was not, nor was apoptosis induced (Figure 2). Thus, collectively, our data show that one of the effects of CO is to inhibit the maturation of pre-OCs induced by RANKL.

The regulatory mechanisms by which RANKL and its intracellular signaling pathways enable pre-OCs to differentiate have been extensively studied [8,35,36]. RANKL binds to RANK expressed on the plasma membrane of OC precursors [25,27,37-41], which then activates an intricate signaling cascade that includes NF-κB, c-fos, c-jun, MAPKs, and NFATc1 [36,42]. Support for the role of this pathway in osteoclastogenesis comes from a previous study in which inhibition of c-fos expression in mice blocked OC differentiation and caused osteopetrosis [39]. IκB-α binds to NF-κB in the cytoplasm, thus maintaining the protein in an inactive form and tightly regulating its transcriptional activity. In our study, CO had no effect on the NF-κB (IκB-α) pathway, as shown in a western blot analysis of IκB-α expression and measurements of RANK mRNA levels (Figure 3A, 4A). However, CO did inhibit the maturation of RANKL-treated RAW 264.7 cells. So what is the nature of the signaling pathway involved in CO-inhibited osteoclastogenesis? Given the CO-mediated reductions in c-jun and JNK phosphorylation and c-fos expression in RANKL-treated RAW 264.7 cells (Figure 3B, Figure 3C), CO could inhibit RANKL-induced osteoclastogenesis by deactivating AP-1, thus down-regulating c-fos, a component of this complex. The activity of AP-1 (containing c-fos) is crucial for the auto-amplification of NFATc1 [43], which is induced and activated by RANKL signaling during the terminal differentiation of OCs [36,42]. Among the OC-specific genes regulated by NFATc1 are *Acp5*, *Calcr*, *Cstk*, and *mmp9*[43-45]. In the presence of CO, the mRNA levels of all four genes was significantly reduced (Figure 4B). In addition, CO inhibited the translation of NFATc1 (Figure 4C). Together, these findings suggest that CO acts on the MAPK pathway to inhibit osteoclastogenesis by RAW 264.7 cells induced to differentiate by stimulation with RANKL.

Interactomics yields deep insights into the molecular mechanism of diseases and their intracellular signaling pathways [28] but, to the best of our knowledge, it has not been used to profile osteoclastogenesis. In this study, a STRING database analysis of RANKL-induced osteoclastogenesis established a PPI map with three clusters (Figure 5A). Cluster I is a highly symmetric, connected protein cluster with strong interactions. It contains Fos, Jun, retinoblastoma binding protein 7 (RBBP7), nuclear factor of kappa light polypeptide gene enhancer in B-cells inhibitor, beta (NFKBIB), CREB binding protein (CREBBP, CBP), peroxisome proliferator-activated receptor gamma (PPARG), NFATc1, and MAPKs. RBBBP7, also called RbAp46, is a histone H4 binding protein that binds to the c-fos transcriptional activation site to inhibit cell mitosis [46]. We found that the prevention of differentiation by RANKL-treated RAW264.7 cells was accompanied by a decrease in c-fos expression, which suggests that CO alters the action of RBBBP7 on c-fos during osteoclastogenesis. Another cluster I protein, the nuclear receptor PPARG, is pivotal for adipogenesis and interacts with histone deacetylase 3 (HDAC3) [47], contained in cluster II along with HDAC7. HDACs catalyze the removal of acetyl groups from an ϵ-N-acetyl lysine amino acid on histones, which are contained in cluster III. In a previous report the use of shRNA to inhibit HDAC3 expression also inhibited OC formation whereas similar inhibition of HDAC7 accelerated OC differentiation [48]. These findings suggest that the balance between HDAC3 and HDAC7 expression decides the fate of pre-OCs exposed to CO. Among the cluster III histones are the histone H2A family (H2AF) members X and Z (H2AFX, H2AFZ), and histone H3 family member 3A (H3F3A). Histones are highly alkylated and comprise the major protein component of chromatin. The epigenetic regulation of histones by methylation and acetylation may provide regulatory control of OC differentiation. For example, the expression of NFATc1 induced by RANKL is associated with the demethylation of trimethylated histone H3 lysine 4 and lysine 27 (H3K4me3, H3K27me3) [49].

Proteins identified as controlling hubs are major, central proteins in PPI networks. As shown in Figure 5A, c-Jun strongly interacted with other proteins in cluster I, such as jnk1 and jnk2. Furthermore, this cluster interacts with MAP3K4 (also called MTK1), a protein in the MAPK pathway, through c-Jun. Abell et al. [50] showed that MP3K4 regulates jnk1 and jnk2 to control the activity of histone acetyltransferase. It also controls CBP activity in trophoblast stem cells during the epithelial-mesenchymal transition. In our interactomics analysis, MAP3K4 was designated as a hub protein that interacts with c-Jun, thereby controlling the interaction between CBP and HDAC3 during OC differentiation. PPI maps derived using the IPA software have been widely used to gain insight into molecular interactions, signaling pathways, and pathogenesis [51]. One of the advantages of this approach is data from a limited number of experiments can be analyzed. We therefore took advantage of this method to obtain a global understanding of the signaling pathways that are activated during osteoclastogenesis in the presence of CO (Figure 5B). Our results showed that CO significantly inhibited the expression of the transcriptional factors c-JUN and c-FOS, the protein partners of AP-1 (Figure 3C), which suggests their involvement in the CO-mediated blockade of OC differentiation. JNK1 and JNK2, two proteins controlled by MAP2K4, were shown to interact with JUND and thereby alter the transcriptional activity of JUN. The IPA PPI showed that in RANKL-treated RAW264.7 cells exposed to CO, MAP2K4, a downstream regulator of MAP3K4 [52], interacts with IκB-α and P38.

## Conclusions

In this study, a low concentration of CO (250 ppm) was shown to inhibit osteoclastogenesis in RANKL-treated RAW264.7 cells. An interactome identifying the PPI network involved in the observed effects allows the following conclusions. First, proteins that function as signal transducers, enzymes, and epigenetic regulators are significantly affected by CO during RANKL-induced osteoclastogenesis. Second, CO inhibits osteoclastogenesis through the MAPK signaling pathway. Third, STRING predicted that MAP3K4 is a major controlling hub protein. Fourth, the interactomics software IPA not only predicted a critical role for MAP2K4 but also identified MAP3K4 as a hub protein. Fifth, STRING and IPA provide overlapping, complementary information. While the data obtained with these tools are similar, the latter provided both a more complete PPI and an easier approach to understanding the behavior of each protein during CO-regulated osteoclastogenesis. Our research offers new data and thus new insights into CO-regulated osteoclastogenesis. However, further detailed investigations into the molecular mechanisms underlying this process are needed.

## Methods

Methylene blue solution, sarkosyl, and the TRAP staining and leukocyte acid phosphatase assay kits were purchased from Sigma (St. Louis, MO, USA). Recombinant RANKL protein was obtained from PeproTech (Rocky Hill, NJ, USA). Anti-IκB antibody was obtained from Biolegend (San Diego, CA, USA). Antibodies for phospho-p38, phospho-JNK, phospho-ERK, phospho-c-jun, and c-fos were purchased from GeneTex (Irvine, CA, USA). Anti-RANKL and anti-actin antibodies were obtained from Abcam (Cambridge, UK), and Sigma, respectively.

### Cell culture

RAW 264.7 cells were cultured in DMEM with 10% FCS, 2 mM l-glutamine, 10 units penicillin/mL, and 10 μg streptomycin/mL at 37°C in a 5% CO_2_ humidified incubator. RAW 264.7 cells were transferred to 100-mm dishes when they reached 80% confluence and further grown in the culture medium.

### Cell proliferation assay

RAW 264.7 cells were grown in 24-well plates (10^4^ cells/mL per well). The medium was removed at day 3 and the cells were stained with methylene blue solution (0.3 mL per well) at room temperature for 30 min, followed by three to four washes with Milli-Q water. The cells were then air dried and dissolved overnight in 1% sarkosyl in phosphate-buffered saline (PBS, 0.3 mL/well). The cell solution was transferred to a 96-well plate (0.1 mL/well), and the absorbance was read at 540 nm using an ELISA plate spectrophotometer.

### In vitro OC differentiation

Cells (10^4^ per well) were grown in 24-well plates and exposed to RANKL (5–20 ng/mL) for 120 h to induce OC formation [24]. These cells were fixed and then stained for TRAP expression using a TRAP staining kit according to the manufacturer’s protocol. The cells were observed using an Olympus BX51 microscope equipped with DP controller (ver. 3.3.1.292). Those with more than three nuclei were identified as TRAP (+) OCs.

### CO exposure

The cells were incubated at 37°C in the presence or absence of 5% CO_2_. The gas was prepared by using an air mixer to dilute an initial concentration of 1% (10,000 ppm) in compressed air with fresh air to obtain a final concentration of 250 ppm, which was then delivered into the incubator. A CO analyzer (Siemens Ultramat 23, Germany) with a sensitivity of 10–600 ppm was used to measure CO levels.

### Immunofluorescence analysis

F-actin rings were detected as described previously [53]. Briefly, the cells were fixed with 4% paraformaldehyde, permeabilized with 0.5% Triton X-100 in PBS, and incubated with an anti-actin antibody at 4°C overnight. After a PBS wash, the cells were incubated with FITC-conjugated secondary antibody for 30 min at 37°C and then analyzed using a Olympus BX51 microscope equipped with a DP controller (version 3.3.1.292).

### Pit formation assay

RAW264.7 cells (1 × 10^3^) were co-cultured with RANKL on dentin discs (Immunodiagnostic Systems Inc., Fountain Hills, AR, USA) in a 96-well plate for 72 h in the presence (CO group) or absence (air group) of CO. Typically, three discs were prepared per group. To observe the areas containing resorption lacunae, the cells were removed the discs were incubated in 0.25 M ammonium hydroxide, washed with distilled water, and then stained with 0.5% (wt/vol) toluidine blue. The resorbed areas were imaged using a reflective optical microscope (LSM 510, Zeiss).

### Western blotting analyses

The cells were washed twice with PBS and protein extracts of the nucleus and cytosol were prepared using a ProteoJET cytoplasmic and nuclear protein extraction kit (Fermentas, Glen Burnie, MD, USA). The extracts were centrifuged at 10,000 × *g* for 5 min after which the supernatants were collected and treated with protease inhibitors. The protein concentration was determined using the Bradford protein assay. The extracts (20 μg) were then dissolved in 6× Laemmli sample loading buffer, boiled for 10 min, and subjected to SDS-PAGE on a 10% gel. The proteins in the gel were electrotransferred onto polyvinylidene fluoride membrane (Millipore) with a semi-dry transfer unit at 20 V for 30 min. After a blocking step with 5% skim milk in Tris-buffered saline (TBS; 20 mM Tris base pH 7.6, 150 mM NaCl) containing 1% Tween-20 at room temperature for 1 h, the membrane was incubated with the primary antibody at room temperature at 4°C overnight. It was then washed three times for 10 min with TBS containing 1% Tween-20, incubated with secondary antibody at room temperature for 1 h, and again washed as before. The immunoblotted protein bands were visualized by chemiluminescence using Immobilon western chemiluminescent HRP substrate (Millipore, Billerica, MA, USA) and X-ray film.

### Real-time quantitative reverse transcription-polymerase chain reaction (qRT-PCR) analysis

Trizol reagent was used to isolate total RNA, which was further eluted with 20 μL of RNase-free water. For cDNA synthesis, 5 μg of total RNA was reverse transcribed at 42°C for 60 min using RevertAid first-strand cDNA synthesis kit (Fermentas). The reaction was terminated by heating at 75°C for 5 min. The sequences of the primers were as follows:

Cathepsin K: 5′–ATGTGGGGGCTCAAGGTTCTG–3′ and 5′–CATATGGGAAAGCATCTTCAGAGT C–3′

TRAP: 5′–AGCAGCCAAGGAGGACTACGTT–3′ and 5′–TCGTTGATGTCGCACAGAGG–3′

Calcitonin receptor: 5′–AGTTGCCCTCTTATGAAGGAGAAG–3′ and 5′–GGAGTGTCGTCCCAGCACAT–3′

MMP-9: 5′–GGAACTCACACGACATCTCCA–3′ and 5′–GAAACTCACACGCCAGAAGAATTT–3′

The Maxima SYBR Green/ROX qPCR master mix kit (Fermentas) was used for all qRT-PCRs. The reactions were carried out in a total volume of 20 μL containing 10 μL of 2× Maxima SYBR Green/ROX qPCR, 1 μM of the primer pair, and 5 μL of cDNA. Thermal cycling parameters were 95°C for 10 min, 40 cycles of 95°C for 15 sec, and 60°C for 60 s. The samples for each PCR were prepared in triplicate. The GAPDH gene was used as the control. Data sets were analyzed and amplification plots were obtained automatically by the 7500 Fast System Software (Applied Biosystems). The comparative threshold cycle (2^–[delta] Ct^) method, which converts differences of cycle numbers to the ratio of the test gene/control gene, was used to normalize the gene expression levels to GAPDH mRNA.

### PPI network and signaling pathway analysis

Proteins were analyzed for their interactions and signaling pathways using Search Tool for the Retrieval of Interacting Genes/Proteins (STRING) database version 9.0 (http://string.embl.de) and the Ingenuity Pathways Analysis (IPA) software (http://www.ingenuity.com), respectively.

### Statistics

All statistical analyses were performed using the Student’s *t*-test. Significance was defined as p < 0.05.

## Abbreviations

Acp5: Tartrate resistant acid phosphatase; Calcr: Calcitonin receptor; CBP: CREB binding protein; CO: Carbon monoxide; Cstk: Cathepsin K; ERK: Extracellular regulated kinase; H2AFX: H2A histone family, member X; H2AFZ: H2A histone family, member Z; H3F3A: H3 histone, family 3A; H3K27me3: Lysine 27; H3K4me3: Demethylation of trimethylated histone H3 lysine 4; HDAC3: Histone deacetylase 3; HDAC7: Histone deacetylase 7; JNK: Jun N-terminal kinase; MAP3K4: Mitogen-activated protein kinase kinase kinase 4; MAPKs: Mitogen-activated protein kinases; M-CSF: Macrophage colony-stimulating factor; MMP9: Matrix metalloproteinase-9; NF-κB: Receptor activator of nuclear factor-κB; NFKBIB: Nuclear factor of kappa light polypeptide gene enhancer in B-cells inhibitor, beta; OCs: Osteoclasts; OBs: Osteoblasts; PPARG: Peroxisome proliferator-activated receptor gamma; RANKL: Receptor activator of nuclear factor-κB ligand; RBBP7: Retinoblastoma binding protein 7; TNF: Tumor necrosis factor; TRAP: Tartrate-resistant acid phosphatase.

## Competing interests

The authors declared that there are no competing interests.

## Authors’ contributions

The work presented here was carried out as a collaboration between all of the authors. RYP and HKS defined the research theme. FJT and WTC designed the methods and experiments, FJT and GHG carried out the laboratory experiments, analyzed the data, interpreted the results, and wrote the paper. CWH and MJT carried out the STRING and IPA analyses. RYP and JFS co-designed the dispersal and colonization experiments, and worked together on the collection of the data and their interpretation. FJT co-designed the experiments, and contributed to discussions of the analyses, interpretation, and presentation. All authors have contributed to, read, and approved of the manuscript.

## Supplementary Material

Additional file 1: Figure 1 CO reduced the number of TRAP(+) cells in bone marrow macrophages. **Air+R**, ordinary incubate condition (37°C, 5% CO2) with MCSF 20 ng and RANKL 20 ng added; **CO+R,** ordinary incubate condition plus CO, MCSF 20 ng and RANKL 20 ng. Click here for file

Additional file 2: Figure 2 Positive control experiment with Calcitonin 30 nM and RANKL 20 ng in RAW cells. Click here for file
