# Supplementary figures and images for: Interactomics profiling of the negative regulatory function of carbon monoxide on RANKL-treated RAW 264.7 cells during osteoclastogenesis
Source: BMC Syst Biol. 2014 May 18;8:57. doi: 10.1186/1752-0509-8-57 (PMC4052347; doi:10.1186/1752-0509-8-57)

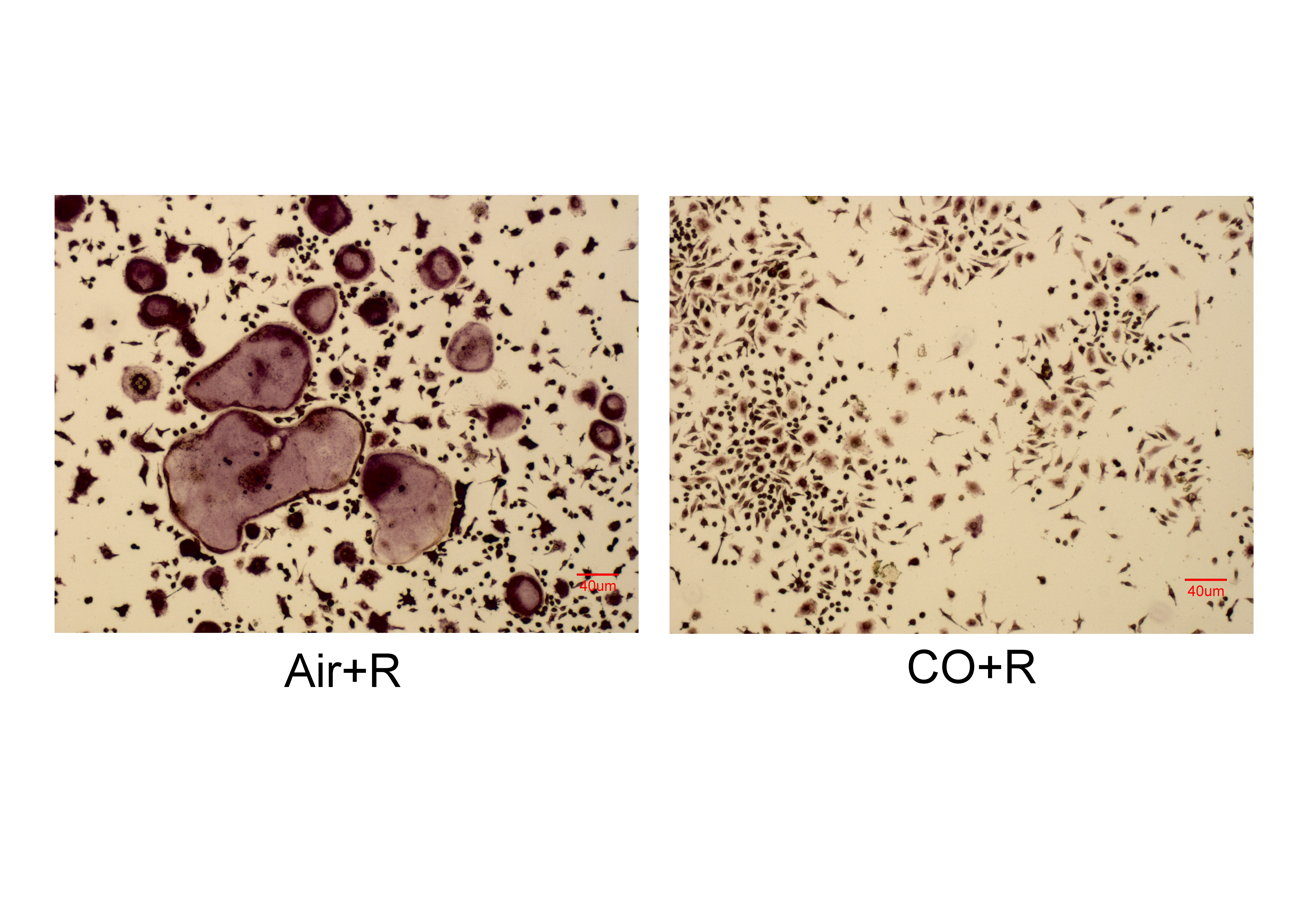

Supplement: Additional file 1: Figure 1 — CO reduced the number of TRAP(+) cells in bone marrow macrophages. Air+R, ordinary incubate condition (37°C, 5% CO2) with MCSF 20 ng and RANKL 20 ng added; CO+R, ordinary incubate condition plus CO, MCSF 20 ng and RANKL 20 ng. [file 1752-0509-8-57-S1.png]

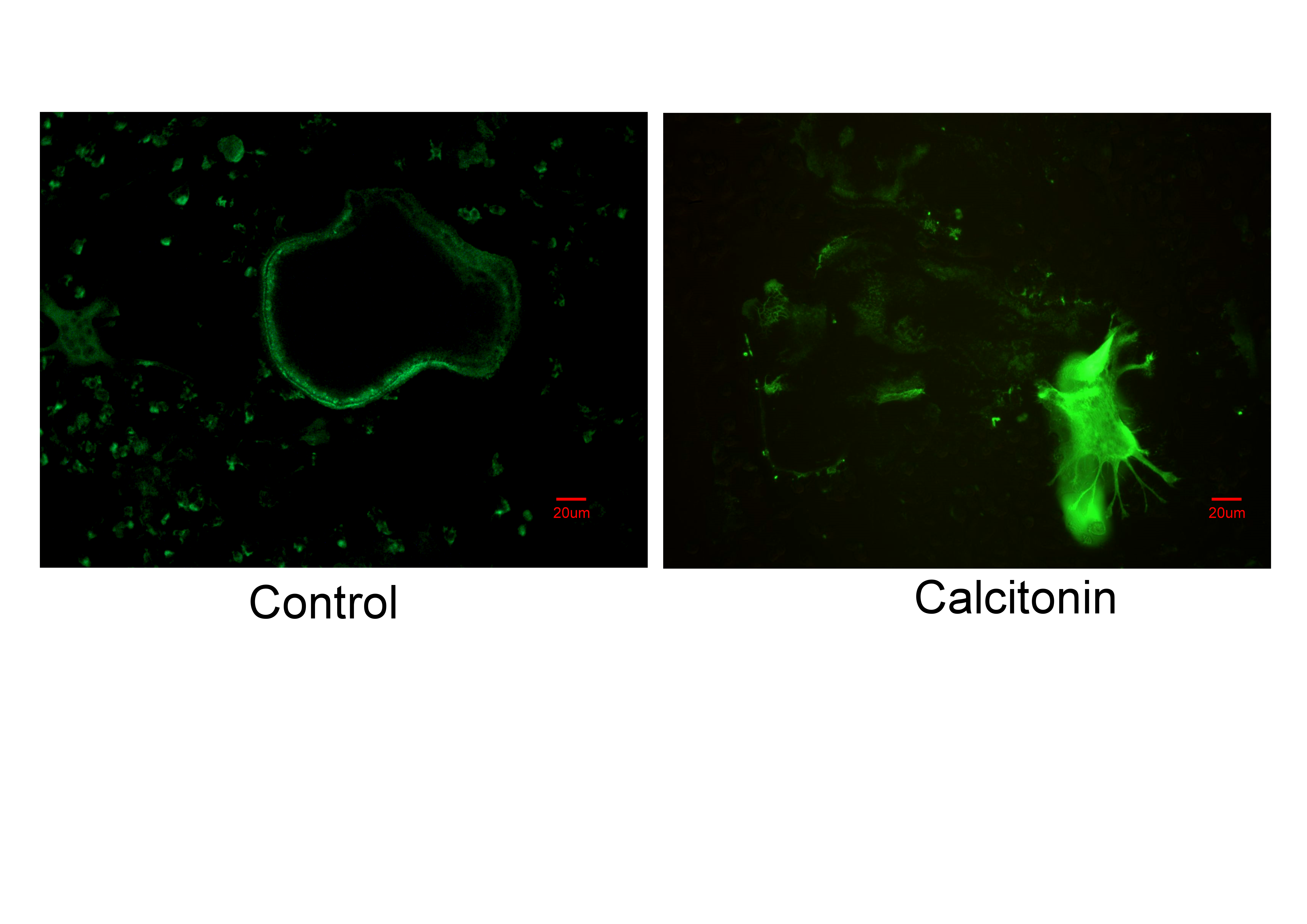

Supplement: Additional file 2: Figure 2 — Positive control experiment with Calcitonin 30 nM and RANKL 20 ng in RAW cells. [file 1752-0509-8-57-S2.png]
